# Molecular epidemiology of clinical filamentous fungi in Qatar beyond *Aspergillus* and *Fusarium* with notes on the rare species

**DOI:** 10.1093/mmy/myac098

**Published:** 2023-01-02

**Authors:** Husam Salah, Jos Houbraken, Teun Boekhout, Muna Almaslamani, Saad J Taj-Aldeen

**Affiliations:** Division of Microbiology, Department of Laboratory Medicine and Pathology, Hamad Medical Corporation, Doha, Qatar; Yeast Research, Westerdijk Fungal Biodiversity Institute, Utrecht, Netherlands; Applied and Industrial Mycology, Westerdijk Fungal Biodiversity Institute, Utrecht, Netherlands; Yeast Research, Westerdijk Fungal Biodiversity Institute, Utrecht, Netherlands; Institute of Biodiversity and Ecosystem Dynamics (IBED), University of Amsterdam, Amsterdam, The Netherlands; Department of Medicine, Hamad Medical Corporation, Doha, Qatar; Division of Microbiology, Department of Laboratory Medicine and Pathology, Hamad Medical Corporation, Doha, Qatar; Department of Biology, College of Science, University of Babylon, Hilla, Iraq

**Keywords:** filamentous fungi, invasive fungal infections, molecular epidemiology, Middle East, Qatar

## Abstract

Due to an increasing number of patients at risk (i.e., those with a highly compromised immune system and/or receiving aggressive chemotherapy treatment), invasive fungal infections (IFI) are increasingly being reported and associated with high mortality rates. *Aspergillus* spp., particularly *A. fumigatus*, is the major cause of IFI caused by filamentous fungi around the world followed by *Fusarium* spp., however, other fungi are emerging as human pathogens. The aim of this study was to explore the epidemiology and prevalence of the non-*Aspergillus* and non-*Fusarium* filamentous fungi in human clinical samples over an 11-year period in Qatar using molecular techniques. We recovered 53 filamentous fungal isolates from patients with various clinical conditions. Most patients were males (75.5%), 9.4% were immunocompromised, 20.7% had IFI, and 11.3% died within 30 days of diagnosis. The fungal isolates were recovered from a variety of clinical samples, including the nasal cavity, wounds, respiratory samples, body fluids, eye, ear, tissue, abscess, and blood specimens. Among the fungi isolated, 49% were dematiaceous fungi, followed by *Mucorales* (30%), with the latter group *Mucorales* being the major cause of IFI (5/11, 45.5%). The current study highlights the epidemiology and spectrum of filamentous fungal genera, other than *Aspergillus* and *Fusarium*, recovered from human clinical samples in Qatar, excluding superficial infections, which can aid in the surveillance of uncommon and emerging mycoses.

## Introduction

The incidence of fungal infections is increasing worldwide. About a billion people are affected with superficial (skin, hair, and nail) fungal infections worldwide.^1^ Life-threatening invasive fungal infections (IFI) affect primarily immunocompromised individuals with neutropenia, cancer, organ transplantation, HIV/AIDS, and those receiving immunosuppressive therapy. Other risk factors associated with serious fungal infections include asthma, chronic obstructive pulmonary disease (COPD), and tuberculosis.^2^ The mortality of IFI exceeds 1.6 million per year on a global scale.^3–5^ Recently, the World Health Organization (WHO) released the first-ever fungal pathogens priority list (WHO-FPPL) which categorizes fungal pathogens based on their public health importance and unmet research needs.^6^ The WHO-FPPL focuses on fungi that might cause invasive acute or subacute systemic infections as well as those which pose treatment and management difficulties. Pathogens were classified into three priority groups (critical, high, and medium). The critical group includes *Cryptococcus neoformans, Candida auris, Aspergillus fumigatus*, and *C. albicans. Nakaseomyces glabrata* (*C. glabrata*), *Histoplasma* spp., eumycetoma causative agents, *Mucorales, Fusarium* spp., *C. tropicalis*, and *C. parapsilosis* were assigned to the high group. *Scedosporium* spp., *Lomentospora prolificans, Coccidioides* spp., *Pichia kudriavzeveii* (*C. krusei*), *Cryptococcus gattii, Talaromyces marneffei, Pneumocystis jirovecii*, and *Paracoccidioides* spp. are pathogens in the medium category.

Filamentous fungi other than *Aspergillus* and *Fusarium* that cause human disease are emerging.^7–10^ These are clinically difficult to distinguish from aspergillosis and fusariosis. Moreover, many of these fungi are intrinsically resistant to the commonly used antifungal drugs, making them difficult to treat and this may lead to high mortality rates.^9–12^ The epidemiology of non-*Aspergillus* filamentous fungal infections varies geographically.^13,14^ For example, *Fusarium* is the second most common filamentous fungus causing human infections in the United States and Europe,^8,15^ whereas in Australia, infections caused by *Scedosporium* spp. were found to be more common than those caused by *Fusarium* spp.^13^

Many expatriates from high-risk regions of the world, mainly Southeast Asia, make up Qatar's population and that may explain the diverse fungal genera recovered from susceptible individuals. A few studies on the epidemiology of filamentous fungal infections have been published from the Middle East.^16,17^ In addition, several studies on fungal diseases in Qatar have been published, including mucormycosis,^18–20^*Candida* infections,^21–25^ fusariosis,^26,27^ and aspergillosis,^28–30^ however, most of these studies were case reports. Furthermore, the burden of fungal infections in Qatar was estimated by Taj-Aldeen et al. from January 2009 to December 2014.^31^ Except for mucormycosis,^18–20,32–35^ studies from the Middle East reported only a few cases of filamentous fungal infections other than aspergillosis and fusariosis.^36–40^

The current study aimed to investigate the epidemiology of pathogenic filamentous fungi in Qatar other than *Aspergillus* and *Fusarium*, as these genera have been addressed elsewhere,^26,27,30^ using internal transcribed spacer (ITS) region sequences for identification.

## Materials and methods

### Patients and specimens

A total of 53 clinical specimens positive for filamentous fungi belonging to 53 patients were recorded in about 11 years (September 2003–November 2014) (Table 1). These specimens were received from various facilities of the Hamad Medical Corporation (HMC) in addition to primary health centres and private hospitals in Qatar. They were isolated and identified by morphology according to the standard operating procedures of the Microbiology Laboratory at Hamad General Hospital, Qatar.

**Table 1.** Patients’ demographics, clinical data, mortality, and fungi isolated.

	Specimen	Gender/		Clinical		Mortality		Identification	Genbank
S. No	number	age	Origin	data	Histopathology	(30 days)	Specimen type	(ITS)	accession#
1	Q0466	M/63	Pakistan	NA^a^	NA	Alive	Wound tissue	*Aureobasidium mangrovei*	ON387555
2	Q0894	F/23	Qatar	Nasal polyp	Positive (**Proven**)	Alive	Nasal polyp	*Curvularia* sp.	ON387540
3	Q6540	F/54	Syria	Breast cancer	NA (**Proven** by blood)	Died	Blood	*Sarocladium kiliense*	ON387561
4	Q1292	M/41	Qatar	Invasive fungal sinusitis renal transplant	Positive (**Proven**)	Alive	Nasal swab	*Rhizopus oryzae*	ON387607
5	Q0888	M/21	Qatar	Allergic fungal sinusitis	NA	Alive	Nasal polyp	*Curvularia* cf. *buchloes*	ON387527
6	Q0051	M/14	India	Paranasal fungal sinusitis	NA	Alive	Nasal swab	*Curvularia* cf. *buchloes*	ON387526
7	Q0141	M/29	Egypt	Trauma	NA	Alive	Foot tissue	*Lichtheimia hongkongensis*	ON387599
8	Q0268	M/62	Palestine	COPD^b^	NA	Alive	Sputum	*Alternaria alternata*	ON387548
9	Q0518	M/34	Sudan	Eumycetoma (Madura foot)	Positive (**Proven**)	Alive	Pus swab (foot)	*Acremonium breve*	ON387562
10	Q0767	M/26	Burma	NA	NA	Alive	Plate culture	*Lichtheimia hongkongensis*	ON387600
11	Q1088	M/55	Qatar	Liver transplant	Positive from leg ulcer (**Proven**)	Alive	BAL^d^	*Mucor indicus*	ON387609
12	Q0947	M/26	Nepal	Corneal abscess	NA	Alive	Corneal scrapings	*Dothichiza pimprina*	ON387551
13	Q0286	F/59	Qatar	Breast cancer, on chemotherapy, fungal encephalitis	Positive (**Proven**)	Died	Brain abscess	*Rhinocladiella mackenziei*	ON387593
14	Q1003	M/31	India	Infected leg fracture	Positive (**Proven**)	Alive	Leg tissue	*Rhizopus microsporus*	ON387603
15	Q1314	M/16	India	Allergic fungal sinusitis	Positive (allergic)	Alive	Nasal tissue	*Curvularia* sp.	ON387534
16	Q0210	M/7	Qatar	Obstructive jaundice	NA	Alive	Gastric aspirate	*Exophiala dermatitidis*	ON387592
17	Q1325	M/78	Palestine	Pneumonia	NA	Died	Peritoneal fluid	*Curvularia* sp.	ON387533
18	Q1293	M/73	Qatar	Diabetic foot	NA	Alive	Toe tissue	*Rhizopus oryzae*	ON387608
19	Q0748	M/39	Sudan	Eye discharge	NA	Alive	Eye swab	*Curvularia* sp.	ON387541
20	Q0784	M/22	Qatar	Fungal sinusitis	Positive (**Proven**)	Alive	Nasal tissue	*Curvularia* sp.	ON387528
21	Q0852	M/43	Philippines	Dyspnea	Negative	Alive	Bronchial wash	*Paecilomyces formosus*	ON387591
22	Q1036	M/23	Qatar	Allergic fungal sinusitis	Positive (allergic)	Alive	Nasal tissue	*Curvularia* sp.	ON387544
23	Q7012	M/53	India	Corneal abscess	NA	Alive	Corneal scrapings	*Curvularia lunata*	ON387542
24	Q1343	M/48	Egypt	Trauma	NA	Died	Wound	*Curvularia* sp.	ON387532
25	Q1337	M/36	Oman	Left leg cellulitis	Negative	Alive	Leg tissue	*Rhizopus oryzae*	ON387606
26	Q6551	M/62	Iran	Corneal abscess	NA	Alive	Corneal scrapping	*Curvularia* sp.	ON387529
27	Q0167	M/79	Qatar	Abdominal aortic aneurysm	NA	Died	Bronchial wash	*Lichtheimia* sp.**	-
28	Q0786	M/24	Nepal	NA	NA	Alive	Ear swab	*Scopulariopsis brevicaulis*	ON387563
29	Q1963	M/20	Sri Lanka	Leg fracture	Negative	Alive	Wound swab	*Scedosporium apiospermum*	ON387565
30	Q2374	M/36	Nepal	Trauma	NA	Alive	J-Vac fluid	*Mucor indicus*	ON387610
31	Q1066	F/26	Qatar	NA	NA	Alive	Nasal swab	*Curvularia* sp.	ON387539
32	Q5775	M/34	India	Corneal abscess	NA	Alive	Eye swab	*Subramaniula asteroides*	ON387556
33	Q1249	F/58	Sudan	Infected Sternal wound	NA	Alive	Wound tissue	*Curvularia* sp.	ON387531
34	Q0445	M/31	Sudan	Allergic fungal sinusitis	Positive (allergic)	Alive	Nasal tissue	*Curvularia* sp.	ON387543
35	Q4920	M/29	Nepal	Trauma	NA	Alive	Wound swab	*Lichtheimia ornata*	ON387602
36	Q5822	F/30	India	Ear discharge	NA	Alive	Ear swab	*Syncephalastrum monosporum*	ON387612
37	Q6111	F/75	Qatar	Upper respiratory tract infection	NA	Alive	Sputum	*Mucor circinelloides*	ON387611
38	Q1114	F/36	India	Diabetic ketoacidosis, septic shock	NA	Alive	BAL	*Curvularia* sp.	ON387545
39	Q9189	M/26	Nepal	NA	NA	Died	Wound swab	*Lichtheimia* sp.**	-
40	Q0450	M/78	Qatar	Diabetic foot	NA	Alive	Foot tissue	*Scopulariopsis brevicaulis*	ON387564
41	Q0458	M/49	Eritrea	Hemoptysis, chronic cough	NA	Alive	BAL	*Paecilomyces variotii*	ON387590
42	Q1162	M/44	Nepal	Fungal sinusitis	Positive (**Proven**)	Alive	Nasal tissue	*Rhizopus oryzae*	ON387605
43	Q0513	F/32	Qatar	Renal failure	NA	Alive	Peritoneal dialysis fluid	*Quambalaria cyanescens*	ON387595
44	Q0394	M/54	India	AML^c^, cellulitis, below knee amputation	NA	Alive	Foot tissue	*Lichtheimia corymbifera*	ON387601
45	Q0719	F/42	Qatar	NA	NA	Alive	Nose swab	*Alternaria alternata*	ON387549
46	Q0870	M/20	Iran	NA	NA	Alive	Nasal swab	*Trichoderma longibranchiatum*	ON387560
47	Q0926	M/51	India	Trauma	NA	Alive	Tissue (leg wound)	*Rhytidhysteron rufulum*	ON387552
48	Q4037	M/3	Qatar	Trauma	NA	Alive	Thumb wound	*Curvularia* sp.	ON387536
49	Q2296	F/36	Qatar	Pleural effusion	NA	Alive	Pleural fluid	*Quambalaria cyanescens*	ON387598
50	Q1669	F/51	Qatar	NA	NA	Alive	Nasal swab	*Schizophyllum commune*	ON387594
51	Q1687	M/11	Qatar	Allergic fungal sinusitis	NA	Alive	Nasal tissue	*Curvularia* sp.	ON387538
52	Q1783	M/28	Sudan	Invasive fungal sinusitis	Positive (**proven**)	Alive	Nasal tissue	*Curvularia* sp.	ON387535
53	Q1812	F/37	Philippines	Biliary pancreatitis, small bowel perforation, abdominal surgery, systemic mucormycosis	Positive (**proven**)	Alive	Abdominal wall tissue	*Rhizopus microsporus*	ON387604

^a^Data not available.

^b^Chronic obstructive pulmonary disease.

^c^Acute lymphoblastic leukemia.

^d^Broncho–alveolar lavage fluid; *Not identified using ITS sequencing; **Identified by morphology.

### Isolation and identification of fungal pathogens from clinical specimens

Clinical samples were inoculated on Sabouraud dextrose agar (SDA; Difco Laboratories, Detroit, MI) with chloramphenicol (SDA), and SDA without antibiotics. Blood cultures were performed using Bactec FX automated Blood culture system (BD Diagnostic, Franklin Lakes, New Jersey, United States). Culture plates were incubated at 26 °C and 37 °C and were observed daily for growth up to 10 days except for dermatological specimens which were incubated up to 3 weeks. The isolates were harvested in glycerol cryo-tubes (Mast Diagnostics, UK) and stored at −70 °C until use.

### Molecular identification

#### DNA extraction

All isolates were sub-cultured on homemade oatmeal agar (OA)^41^ and incubated for 5 days at 28 °C prior to DNA extraction. DNA was extracted using PrepMan Ultra sample preparation reagent (Applied Biosystems, Foster City, USA) according to the manufacturer's instructions. In short, a loop full of mycelium taken from the edge of the colonies was suspended in 100 μl PrepMan lysis solution in 2 ml sterile screw-cap microcentrifuge tubes and vortexed for 10–30 s. The mixture was heated at 100 °C in a heat block for 10 min and then centrifuged at 12 000 rpm for 2 min. A total of 50 μl supernatant containing the fungal DNA was transferred to another microcentrifuge tube. The DNA extracts were pipetted to a 96-well plate and the PCR master mix was added using a semi-automated multichannel pipetting robot (Integra Viaflo 96, INTEGRA Biosciences, Switzerland).

#### PCR and sequencing

The ITS region was amplified using the forward primer ITS5 (GGAAGTAAAAGTCGTAACAAGG)^42^ and reverse primer ITS4 (TCCTCCGCTTATTGATATGC).^43^ The PCR mixture per sample contained 6.6 μl of sterile water, 1.25 μl 10x Taq buffer, 1 μl dNTPs mix, 0.63 μl dimethylsulfoxide (DMSO), 0.25 μl of forward and reverse primers, and 0.06 μl Taq polymerase, resulting in a total volume of 10.04 μl. The PCR reactions were performed using the following conditions; an initial denaturation at 95 °C for 5 min, 35 cycles of denaturation at 95 °C for 30 s, 35 cycles of annealing at 55 °C for 45 s, 35 cycles extension at 72 °C for 70 s and finally a step of final extension at 72 °C for 10 min. The PCR products were kept on hold at 10 °C. The sequencing PCR reactions were performed using ABI PrismH Big Dye Terminator Reaction Kit v3.0 (Applied Biosystems, Inc., Foster City, CA, USA) and sequences were obtained with an ABI PRISM™ 3100 Genetic Analyzer (Applied Biosystems, Inc., Foster City, CA, USA) as mentioned previously.^26^ A consensus sequence was generated by combining the forward and the reverse read in the software packages Seqman and Editseq from the Lasergene package (DNAStar Inc., Madison, WI). A homology search with the generated consensus sequences was performed using the Basic Local Alignment Search Tool (BLAST) of the NCBI database.^44^

#### Phylogenetic analysis

To confirm the identification of isolates, a phylogenetic tree based on ITS sequences was constructed. The sequences were aligned using MAFFT v. 7.490 online version (https://mafft.cbrc.jp/alignment/server/). The aligned sequences were manually edited in Molecular Evolutionary Genetics Analysis version 7 software (MEGA7) and phylogenetic trees were inferred using the Maximum Likelihood method based on the Tamura 3-parameter model and 1000 bootstrap replications in MEGA7.^45^

## Results

### Patients characteristics

Filamentous fungi, other than *Aspergillus* and *Fusarium*, were isolated from 53 patients, 40 (75.5%) of them were males, with various clinical conditions. Their ages ranged from 3 to 79 years (median of 41 years), and five patients (9.4%) were <18 years old. Patients originated from 14 countries including the Middle East (*n* = 32, 60.3%), Southeast Asia (*n* = 20, 37.7%), and one patient from Eritrea (*n* = 1, 9%). The clinical presentations and the underlying conditions for 28 patients are presented in (Table 1). Patients included 5 (9.4%) immunocompromised individuals, 11 patients (20.7%) had proven IFI, and 6 (11.3%) died within 30 days after diagnosis. Risk factors were available for 25 patients and included trauma (*n* = 8, 15%), diabetes mellitus (*n* = 3, 5.7%), surgery (*n* = 4, 7.5%), cancer (*n* = 2, 3.8%), soft organ transplantations (SOT) (*n* = 2, 3.8%), and 1 case (1.9%) each of hematological malignancy, burn, COPD, and renal failure (Table 1).

### Clinical specimens

**Figure 1.**
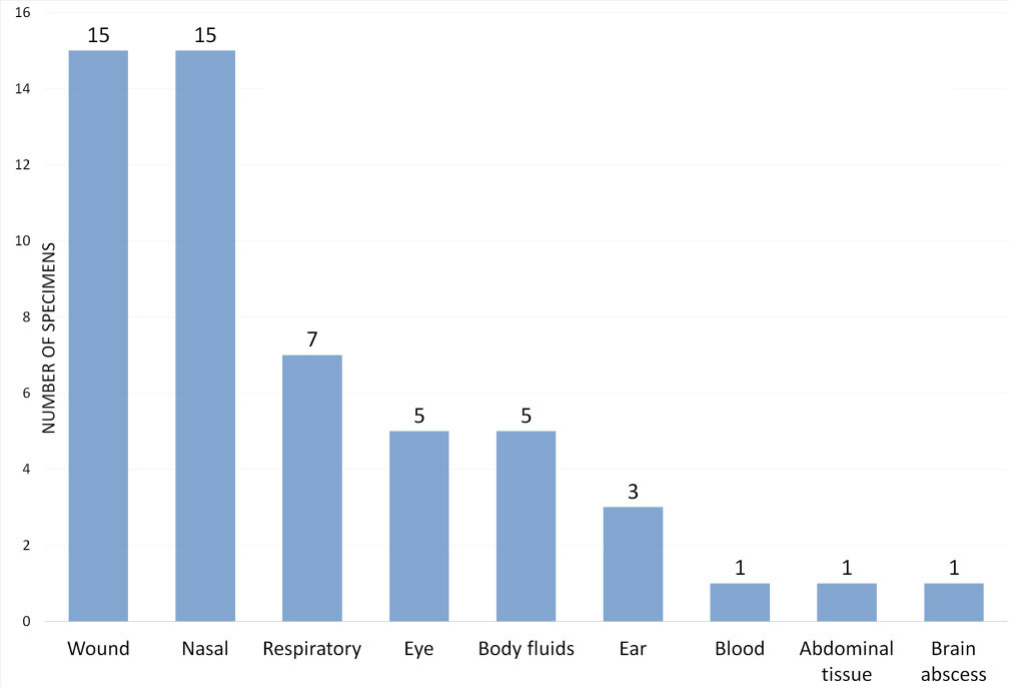
Distribution of clinical samples.

Fungi were recovered from various clinical specimens including nasal specimens (*n* = 15, 28.3%), wounds (*n* = 15, 28.3%), respiratory specimens (*n* = 7, 13.2%), body fluids (*n* = 5, 9.4%), eye (*n* = 5, 9.4%), ear swabs (*n* = 2, 3.8%), and one isolate each from an abdominal tissue, brain abscess, blood, and a clinical specimen that was received from an external facility for fungal identification with unknown specimen source (Table 3 and Fig. 1).

#### Isolated fungi

**Figure 2.**
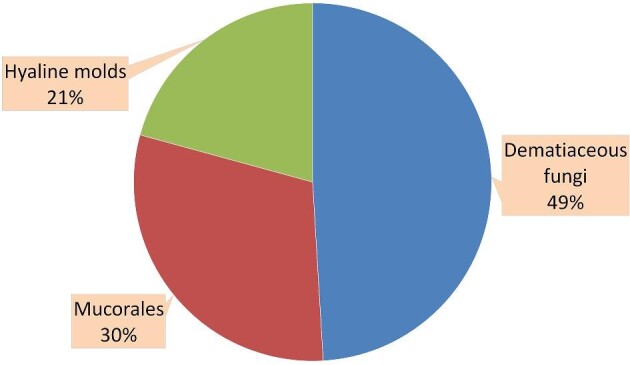
Distribution of fungal isolates.

The molecular identification of clinical fungi using ITS sequencing resulted in 51 isolates that belonged to 20 fungal genera (Table 1). The isolates were deposited to the Genbank database and their accession numbers are listed in Table 1. Two isolates were not identified due to poor sequence data, they were identified by morphological features as *Lichtheimia* species. Overall, dematiaceous fungi were the most isolated fungi in our study (26/53, 49%), followed by *Mucorales* (16/53, 30%) and other hyaline fungi (11/53, 21%) (Fig. 2). Most of the dematiaceous fungi (*n* = 18/26, 69%) belonged to the genus *Curvularia* whereas *Rhizopus* and *Lichtheimia* were the most frequently isolated genera in *Mucorales*, both (6/16, 37.5%).

**Figure 3.**
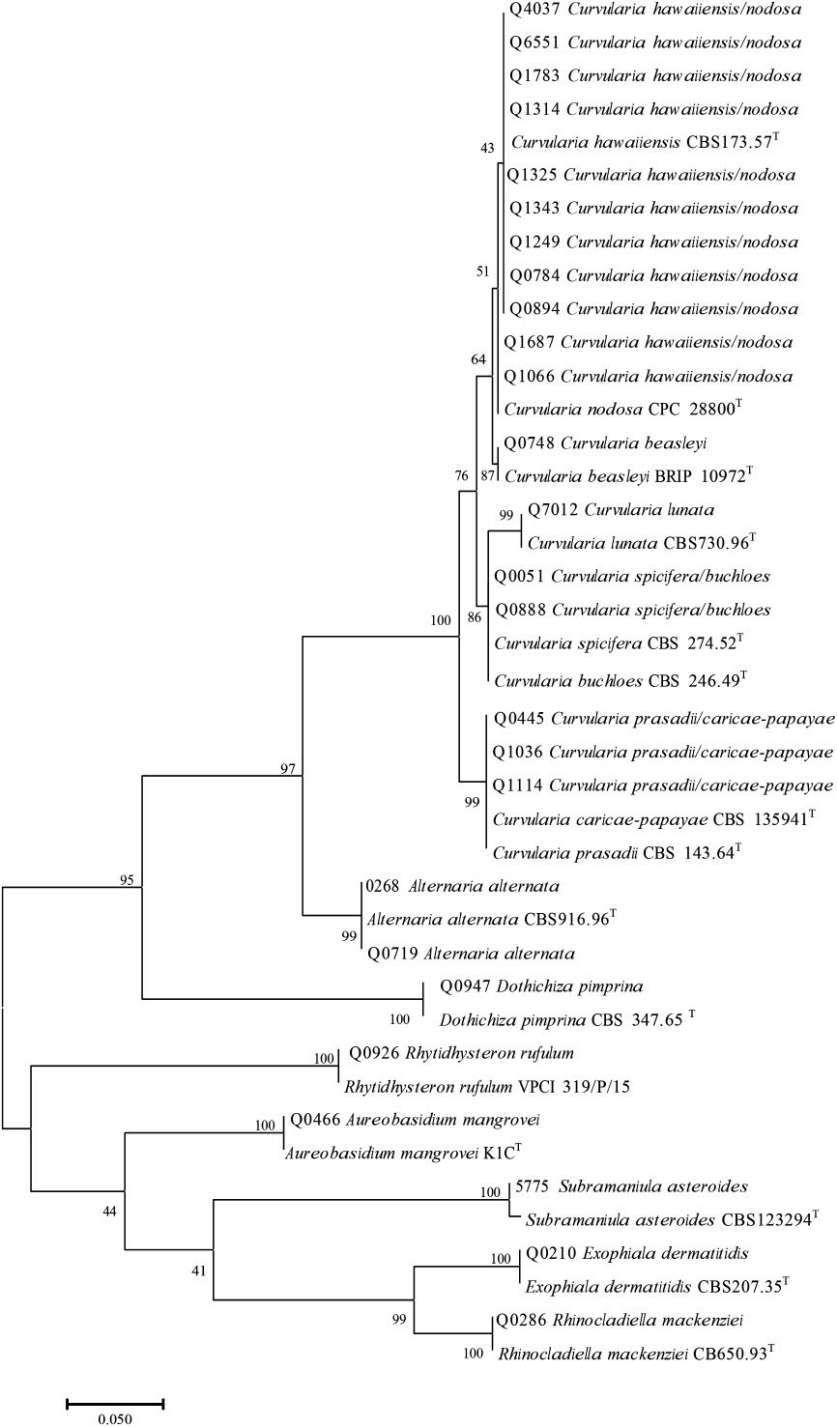
Phylogenetic tree of dematiaceous fungi generated by Maximum Likelihood (ML) based on internal transcribed spacer (ITS) gene. ^T^Type strain.

**Figure 4.**
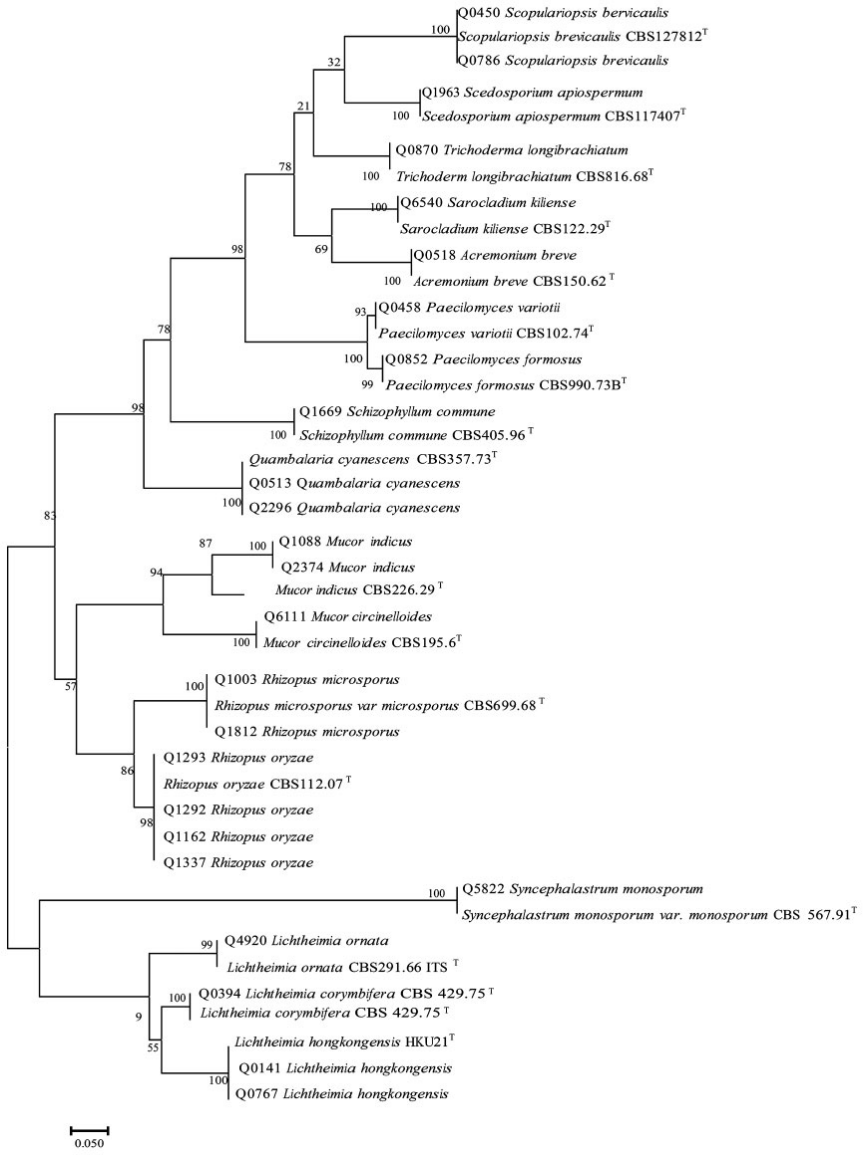
Phylogenetic tree of other filamentous fungi generated by Maximum Likelihood (ML) based on internal transcribed spacer (ITS) gene. ^T^ Type strain.

To confirm the identifications, phylogenetic trees were inferred based on the ITS sequences including type strains (Figs. 3 and 4). All isolates clustered with their corresponding type strains. However, most *Curvularia* species could not be sufficiently separated using ITS sequences. These included *C. hawaiiensis*/*C. nodosa, C. spicifera*/*C. buchloes*, and *C. prasadii*/*C. caricae-papayae* (Fig. 4). Except for two isolates, all had identical ITS sequences with more than one type strain. The isolates Q0051 and Q0888 showed 100% identity with the type strain *C. buchloes* CBS 246.49 and 99% identity with *C. spicifera* CBS 274.52 including one gap. Therefore, both isolates were identified as *Curvularia* cf. *buchloes*.

#### Invasive fungal infections

A total of 11 patients (21%) had proven IFI caused by *Rhizopus* spp. (4/11, 36%), *Curvularia* spp. (3/11, 27%), *Acremonium breve* (1/11, 9%), *Sarocladium kiliense* (1/11, 9%), *Mucor indicus* (1/11, 9%), and *Rhinocladiella mackenziei* (1/11, 9%) (Table 1). These fungi were mostly isolated from nasal specimens (5/11, 45%) and one specimen each from blood, bronchoalveolar lavage (BAL), abdominal tissue, wound tissue, foot pus, and brain abscess. *Mucorales* were the major cause of IFI (5/11, 45%) followed by dematiaceous fungi (4/11, 36%). Among the *Mucorales*, 4/5 were *Rhizopus* spp. and one was *Mucor indicus*. The dematiaceous fungi that caused IFI were *Curvularia* spp. (*n* = 3) and *R. mackenziei* (*n* = 1). We detected a rare fatal case of fungemia caused by *S. kiliense* in a patient with breast cancer. *Acremonium* spp. was recovered from a wound swab of a Sudanese patient who was diagnosed with eumycetoma (Madura foot) and this was confirmed by histopathology. The risk factors associated with IFI are shown in Table 2. They included SOT (*n* = 3), cancer (*n* = 2), abdominal surgery (*n* = 1), and trauma (*n* = 1).

**Table 2.** Risk factors associated with invasive fungal infections (IFI)

Group of fungi	Organism (*n*)	Risk factor	Specimen
*Mucorales* (5)	*Mucor indicus* (1)	Liver transplant	BAL^a^
	*Rhizopus oryzae* (2)	Renal transplant	Nasal
		NA^b^	Nasal
	*Rhizopus microsporus* (2)	Abdominal surgery	Abdominal tissue
		Fracture	Wound tissue
Dematiaceous fungi (4)	*Curvularia* spp. (3)	NA	Nasal
		NA	Nasal
		NA	Nasal
	*Rhinocladiella mackenziei* (1)	Breast cancer	Brain abscess
Hyaline fungi (2)	*Sarocladium kiliense* (1)	Breast cancer	Blood
	*Acremonium breve* (1)	NA	Foot pus

^a^Broncho–alveolar lavage fluid.

^b^Data not available.

**Table 3.** Distribution of fungal isolates and type of clinical specimen.

	Isolate (*n*)	Wound	Nasal	Respiratory	Eye	Body fluid	Ear	Blood	Abdominal tissue	Brain abscess	Unknown
**Dematiaceous fungi (*n* = 26)**											
	*Alternaria alternata* (2)		1	1							
	*Aureobasidium* sp. (1)	1									
	*Curvularia* sp. (18)	3	10	1	3	1					
	*Dothichiza pimprina* (1)				1						
	*Exophiala dermatitidis* (1)					1					
	*Rhinocladiella mackenziei* (1)									1	
	*Rhytidhysteron rufulum* (1)	1									
	*Subramaniula asteroides* (1)				1						
***Mucorales* (*n* = 16)**											
	*Rhizopus* sp. (6)	3	2						1		
	*Lichtheimia* spp. (6)	4		1							1
	*Mucor* sp. (3)			2		1					
	*Syncephalastrum* sp. (1)						1				
**Hyaline fungi (*n* = 11)**											
	*Quambalaria cyanescens* (2)					2					
	*Sarocladium kiliense* (1)							1			
	*Acremonium breve* (1)	1									
	*Paecilomyces variotii* (2)			2							
	*Scopulariopsis brevicaulis* (2)	1					1				
	*Scedosporium apiospermum* (1)	1									
	*Trichoderma* sp. (1)		1								
	*Schizophyllum commune* (1)		1								

#### Rare infections

We recovered clinical isolates of several fungal genera that are rarely encountered as human pathogens. However, these fungi could not be identified as infection-causing or colonizing agents. *Aureobasidium* spp., a black yeast-like fungus, was isolated from a wound tissue of a patient with an unknown clinical condition. Moreover, we have identified *Quambalaria cyanescens* isolates from two patients; one isolated from a peritoneal dialysis fluid of a patient with renal failure, and the other from a pleural fluid of a patient with unknown underlying disease. *Subramaniula asteroides* was isolated from an eye swab of a patient with a corneal abscess whose underlying condition was unknown. Furthermore, *Exophiala dermatitidis* was isolated from a gastric aspirate of a patient with obstructive jaundice. *Paecilomyces* spp. was isolated from BAL fluid of two patients. The underlying conditions of those patients were unknown. Another very rare species, *Dothichiza pimprina*, was isolated from a corneal scraping of a 26-year-old male with no clinical information mentioned. No data were available to confirm that these fungi were the etiological agents of infection.

## Discussion

The epidemiology of filamentous fungal diseases in Qatar is examined in this 11-year retrospective study, excluding aspergillosis and fusariosis, which have already been covered in earlier publications.^26,30,46^ In a previous study, Taj-Aldeen et al. reported the burden of fungal infections in Qatar that were caused by species of *Candida, Aspergillus, Fusarium, Mucorales, Cryptococcus neoformans*, and *Pneumocystis* over a 5-year period (2009–2014).^31^ Their estimates were based on patients’ data retrieved from the microbiology laboratory database. The authors calculated the burden of fungal infections per 100 000 population for candidemia (15.4), *Candida* peritonitis (8.02), intraocular candidiasis (2.05), *Candida* vaginitis (3506), oral/esophageal candidiasis (6.52), cryptococcal meningitis (0.43), *Pneumocystis* pneumonia (0.8), mucormycosis (1.23), fusariosis (1.68), *Aspergillus* ear infections (23.3), onychomycosis (14.8), and rhinosinusitis (2.3).^31^ However, mycoses caused by other filamentous fungi were not estimated and the molecular identification of the etiological agents was not provided. Previously, we published on the molecular epidemiology and antifungal susceptibility patterns of *Aspergillus*^30^ and *Fusarium*^26,27^ species obtained from patients’ samples in Qatar. In the current study, we present the molecular epidemiology of other filamentous fungi using molecular methods for more accurate identification and to better understand the molecular diversity of fungal pathogens. In general, we were able to identify most isolates using sequencing of the ITS region, except two *Curvularia* isolates (*C. hawaiiensis*/*C. nodosa*, and *C. prasadii*/*C. caricae-papayae*) that could not be sufficiently separated using the ITS sequencing only, and were, therefore, identified up to genus level. Sequencing of the glyceraldehyde-3-phosphate dehydrogenase gene along with the ITS region is generally recommended for accurate identification of *Curvularia* species.^47^

Filamentous fungi were isolated from a wide range of patients from various origins, including those coming from regions where fungal diseases are common. This is reflected in the diverse genera of fungi isolated in our study. The 30-day mortality rate in the present study was 11.3%. We were, however, unable to determine whether these infections were the cause of death or whether other risk factors and underlying diseases influenced mortality. For IFI, cancer (18%) and SOT (18%) were the most common risk factors (Table 2). In a recent study from Iran, hematological malignancies and diabetes mellitus were the most prevalent underlying diseases among patients with IFI.^48^ Slavin et al. showed that hematological malignancies (46.7%), diabetes mellitus (23.5%), and chronic pulmonary disease were the most common comorbidities associated with IFI caused by non-*Aspergillus* molds in Australia.^13^

Mucormycosis is becoming more common worldwide,^49–52^ but it is especially prevalent in India and China among patients with uncontrolled diabetes mellitus.^53–56^ However, in a recent study where 600 articles (851 patients) of mucormycosis from January 2000 to January 2017 were analyzed using a literature search, the burden of mucormycosis was found to be slightly higher in Europe (34%) compared with Asia (31%).^57^ The prevalence and distribution of mucoraceous fungi varies geographically. In China, *Mucor* spp. was the most common pathogen causing mucormycosis (54.3%), followed by *Rhizopus* spp. (28.6%).^58^ On the other hand in a study from Europe, *Rhizopus, Mucor* and *Lichtheimia* accounted for 33.7% (58/172), 19.2% (33/172), and 18.6% (32/172) of mucormycosis cases, respectively.^59^*Mucorales* accounted for 30% (16/53) of the fungi isolated in the current study with a predominance of *Rhizopus* and *Lichtheimia* spp. (both 6/16, 37.5%), followed by *Mucor* spp. (3/16, 19%) and *Syncephalastrum* spp. (6%). Moreover, mucormycosis caused 45% (5/11) of the proven IFI in our study and 50% (3/6) of the deceased patients had mucormycosis. The burden of mucormycosis in Qatar was previously estimated to be 1.23/100 000 population.^31^ In neighboring countries, such as Oman, Jordan, Saudi Arabia, Iraq, and Algeria, the burden of mucormycosis was significantly lower with rates of 0.2, 0.02, 0.2, 0.034, and 0.2/100 000 individuals, respectively.^60–62^ In Iran, the rate of mucormycosis was relatively high (9.2/100 000 population),^63^ and this was attributed to the high prevalence of diabetes in the country.^64^

Dematiaceous fungal infections are generally caused by inhalation or inoculation of fungal spores through the skin following trauma.^65,66^ They usually cause superficial infections in immunocompetent patients, but they can rapidly disseminate and cause deep infections in immunocompromised patients.^47,67^ Superficial infections, subcutaneous nodules, and keratitis, are the most common clinical syndromes associated with dematiaceous fungi.^65,66^ In the current study, dematiaceous fungi were the most isolated fungi (49%), and *Curvularia* was the most isolated genus (69%), followed by *Alternaria* (7.7%). Fungal rhinosinusitis was the most common clinical presentation associated with dematiaceous fungi (11/26, 42.3%), followed by keratitis and cutaneous/subcutaneous infections (both 5/26, 19.2%). In a previous multicenter study of 23 transplant centers over 5-year period in the United States, the most common genus was *Alternaria* (32%), followed by *Exophiala* (11%).^68^ In contrast, Schieffelin *et al*. identified 27 cases of phaeohyphomycosis in SOT recipients in which *Exophiala* was the most recovered genus (11/27), followed by *Ochroconis* (3/11) and *Alternaria* (2/11).^67^ Moreover, in studies from India^69^ and Korea,^70^*Exophiala* was the most isolated genus causing phaeohyphomycosis (26% and 71%, respectively). However, we recovered only one case of *Exophiala* from a gastric aspirate specimen of a patient with obstructive jaundice admitted to the intensive care unit (ICU).

*Rhinocladiella mackenziei* is among the common fungi causing cerebral phaeohyphomycosis.^71^ The infection is almost restricted to the Middle East,^72^ however, few cases were reported from other regions as well.^73–75^ We isolated *R. mackenziei* from a brain abscess of a 59-year old female with breast cancer who was undergoing chemotherapy. The fungus resulted in a fatal cerebral phaeohyphomycosis that was proven by histopathology. This case was previously reported by Taj-Aldeen et al.^38^ and considered the second report of *R. mackenziei* from Qatar. The first case was reported in 1993 from brain abscess of a 55-year old male after renal transplant.^72^

We recovered *Rhytidhysteron rufulum* from a specimen of leg wound tissue of a 51 year old male following trauma. This fungus is extremely rare with only six cases reported in the literature. In all, five of them were reported from India^76–79^ and one case from the USA.^80^ Here we report the seventh case of *R. rufulum* from human clinical samples.

In the current study, we isolated *Subramaniula asteroides* from an eye swab of a 34 year old male with corneal abscess. *S. asteroides* is an opportunistic fungal pathogen that rarely cause fungal keratitis and skin infection.^81,82^ This fungus is able to grow at temperatures up to 40 °C.^82^ Previously reported cases of *S. asteroides* infections were endophthalmitis due to trauma in a noninsulin dependent diabetic patient,^83^ fungal rhinosinusitis in a patient without co-morbities,^83^ and a case of fungal keratitis after corneal trauma.^84^ Interestingly, *S. asteroides* was isolated from desert soil in Saudi Arabia.^82^

A corneal scraping sample obtained from a patient with a corneal abscess (Q0947) grew a fungus that had an ITS sequence which was 99.88% identical to the ITS of the isotype of *Dothichiza pimprina* P.N. Mathur & Thirumalachar (CBS 347.65, Genbank: MH858601.1). A review of the literature for this fungus turned up no previous reports. The isotype of *D. pimprina* (CBS 347.65) is the only strain available in GenBank and was isolated from India.^85^

From the ecological perspective, environmental studies showed that *Alternaria* was found to be abundant in the environment of Qatar,^86–90^ whereas *Curvularia* was less frequent.^86,87^ In contrast, our findings showed that *Curvularia* was more prevalent in clinical specimens compared with *Alternaria*. In general, the prevalence of pathogenic melanized fungi in the current study may be attributed to their resistance to extreme environments (such as Qatar's environment), with high temperatures, salinity, dehydration, and solar radiation.^91–93^

Infections caused by *Quambalaria cyanescens* (formerly *Sporothix cyanescens*^94^) are rare. It was previously isolated from immunocompromised^95,96^ and immunocompetent^97–99^ patients with no clinical evidence of infection in most cases. In our study, we isolated *Q. cyanescens* from peritoneal dialysis fluid of a patient with end stage renal disease (ESRD) and a pleural fluid from another patient with a post-surgical pleural effusion. However, there was no clinical evidence to prove infection. 

*Sarocladium kiliense* (formerly *Acremonium kiliense*) was isolated in the current study from blood sample indicating a disseminated disease. Among *Sarocladium, S. kiliense* is associated with the majority of human infections.^100–102^ This fungus has been described as a cause of mycetoma,^103^ keratitis, endophthalmitis, endocarditis, continuous ambulatory peritoneal dialysis-associated peritonitis, and catheter-related fungemia.^104^ In addition, it was also linked to hospital outbreaks.^105,106^

We isolated *Trichoderma* from patient with fungal rhinosinusitis. *Trichoderma* was previously reported from patients with endocarditis, invasive sinusitis, keratitis, cutaneous infections, mediastinitis, peritonitis, pulmonary infections, liver infection, stomatitis, brain abscesses, infection of cardiac implantable electronic device, or disseminated infections.^107^

*Paecilomyces variotti* was obtained from BAL and bronchial wash specimens of two patients with dyspnea and chronic cough, respectively. Rosanne et al. reported that lung was the second most infected site by this fungus (27%) after the peritoneum (33%).^108^ Infections can affect immunocompetent^109,110^ and immunocompromised individuals. Patients with indwelling catheters, in particular, are at greater risk of invasive infection.^108,109^

In the current study, *Acremonium* was isolated from a patient with mycetoma. It was also previously reported to cause keratitis,^111,112^ osteomyelitis,^113,114^ disseminated infection,^115–118^ brain abscess,^119^ pulmonary infections,^120–122^ meningitis,^123–125^ endocarditis,^126^ subcutaneous infections,^127–130^ and peritonitis.^131,132^

*Scedosporium apiospermum* was isolated in our study from a wound swab of a patient following leg fracture. This fungus was reported to cause a wide range of infections in immunocompromised patients,^11,133^ and mostly cause local infections after traumatic inoculation in immunocompetent individuals. There have been several reports of keratitis,^134–138^ corioretinitis,^139^ vertebral osteomyelitis,^140^ post-traumatic brain infection,^141,142^ lymphocutaneous syndrome,^143^ lymphadenitis,^144^ septic arthritis,^145,146^ and post-tuberculosis lung infection^147,148^ caused by *S. apiospermum*.

We isolated *Scopulariopsis brevicaulis* from an ear swab of a patient with unknown clinical condition and a wound swab from another patient with a diabetic foot. *Scopulariopsis* was previously reported from cases of keratitis,^149–153^ otomycosis,^154,155^ onychomycosis,^156^ rhinosinusitis,^157,158^ and disseminated infections.^159^

The majority of reported *Schizophyllum commune* causing human infections appear to be caused by inhalation of fungal spores, resulting in sinusitis^160–162^ and allergic bronchopulmonary mycosis (ABPM).^163^ Mycoses due to *S. commune* is mostly prevalent in Japan compared with other parts of the world.^163^ Ulceration of the palate,^164^ brain abscess,^160,165^ otitis externa,^166,167^ meningitis,^168^ pneumonia,^169^ cutaneous granuloma,^170^ and onychomycosis^171^ caused by *S. commune* have also been reported. In the current study, we report the first case of *S. commune* from Qatar from a 55 year old female with rhinosinusitis. However, no data were available regarding tissue invasion.

## Limitations of the study

Considering that only selected isolates were used, our study may not reflect the exact prevalence of these fungi. Our data, on the other hand, may provide insight into various fungal genera/species involved in human infections in the country. Furthermore, not all patients had complete data on risk factors, underlying illnesses, and clinical manifestations. Additionally, we did not sequence additional genes for species that could not be identified using ITS only. Finally, we were unable to obtain data on antifungal therapy and prophylaxis.

## Conclusion

To conclude, the current study investigated the spectrum of filamentous fungi, other than *Aspergillus* and *Fusarium* that cause human diseases in Qatar. This may help clinicians and infectious diseases specialists to understand the local epidemiology and trends of these infections, particularly those caused by the emerging fungi, which may serve as a guidance for appropriate patients’ management. Identification using molecular methods can aid in accurately determining the species of fungal isolates obtained from clinical samples. However, species cannot be precisely identified using solely ITS sequencing and may require sequencing of additional genes.
