# Correction to: Molecular epidemiology of clinical filamentous fungi in Qatar beyond Aspergillus and Fusarium with notes on the rare species

**DOI:** 10.1093/mmy/myad006

**Published:** 2023-01-24

This is a correction to: Husam Salah, Jos Houbraken, Teun Boekhout, Muna Almaslamani, Saad J Taj-Aldeen, Molecular epidemiology of clinical filamentous fungi in Qatar beyond *Aspergillus* and *Fusarium* with notes on the rare species, *Medical Mycology*, Volume 61, Issue 1, January 2023, myac098, https://doi.org/10.1093/mmy/myac098

In the originally published version of this manuscript, a funding acknowledgment was omitted for Qatar National Library. The manuscript has been updated to include the funding acknowledgement statement: Open Access funding provided by Qatar National Library.

